# MRI characterization of uterine fibroids may predict success of GnRH agonist therapy prior to magnetic resonance focused ultrasound (MRgFUS) treatment

**DOI:** 10.1186/2050-5736-3-S1-O101

**Published:** 2015-06-30

**Authors:** Kelli Bryant, Suzanne LeBlang

**Affiliations:** 1 University MRI, Boca Raton, Florida, United States

## Background/introduction

In managing women with large uterine fibroids, GnRH agonists can be used to reduce fibroid volume and decrease vascularity to enhance treatment outcomes with MRgFUS. GnRH agonists have been shown to reduce fibroid volume by as much as 30-40%. The purpose of this study was to examine the responsiveness of fibroids to pre-treatment with a GnRH agonist in relation to their appearance on T2 weighted images and MRgFUS treatment outcomes.

## Methods

Fifteen women (ages 34-52) with symptomatic uterine fibroids were pre-treated with a GnRH agonist prior to undergoing MRgFUS treatment using the ExAblate device. 7 patients received treatment for 3 months, 4 received treatment for 4 months, 2 received treatment for 6 months and 2 received treatment for an unknown time before the MRgFUS procedure. Other than the 2 patients with unknown GnRH agonist therapy times, all patients obtained their last GnRH agonist injection 3-4 weeks before MRgFUS treatment. These women were selected to receive a GnRH agonist if they possessed fibroids in excess of 10 cm or had a fibroid volume greater than 300 cc. Fibroids were classified by their intensity on T2 weighted images relative to normal myometrium (hypointense, isointense, or hyperintense as well as tissue homogeneity or heterogeneity). A total of 22 fibroids were treated with MRgFUS: 17 hypointense, 3 heterogeneously hypointense, 1 heterogeneously hyperintense and 1 isointense fibroid. Data regarding fibroid volume reduction following GnRH administration, Joules of energy delivered per cc of fibroid tissue ablated and the final non-perfused volume (NPV) were investigated.

## Results and conclusions

The average reduction in size of fibroids following GnRH treatment based on T2 appearance was: hypointense (22%), isointense (44%) heterogeneously hypointense (33%) and heterogeneously hyperintense (40%). The average Joules of energy delivered per sonication was greater for isointense (4550 J) and heterogeneously hyperintense (4200 J) fibroids than for heterogeneously hypointense (3227 J) and hypointense (2654 J) fibroids. Additionally, the volume of tissue ablation per Joule of energy applied was significantly larger for the heterogeneously hypointense (0.066 cm3) and heterogeneously hyperintense (0.057 cm3) than for the hypointense (0.036 cm3) and isointense (0.018 cm3). The NPV per fibroid was greater for heterogeneously hyperintense (85%), heterogeneously hypointense (63%) and hypointense (72%) fibroids than for isointense (32%) fibroids. Fibroid image characteristics may be used to predict the effectiveness of GnRH agonist therapy prior to MRgFUS treatment. While more vascular fibroids require greater energy to treat, they show a more favorable response to pre-treatment with a GnRH agonist in terms of fibroid volume reduction and thermoablative treatment effectiveness than hypointense fibroids.

**Figure 1:**
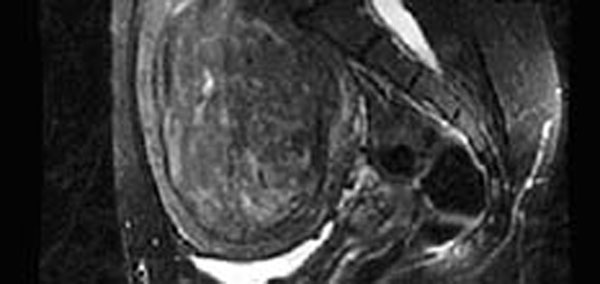
Sagittal T2-weighted image depicting a 471 cc heterogeneously hyperintense fibroid.

**Figure 2:**
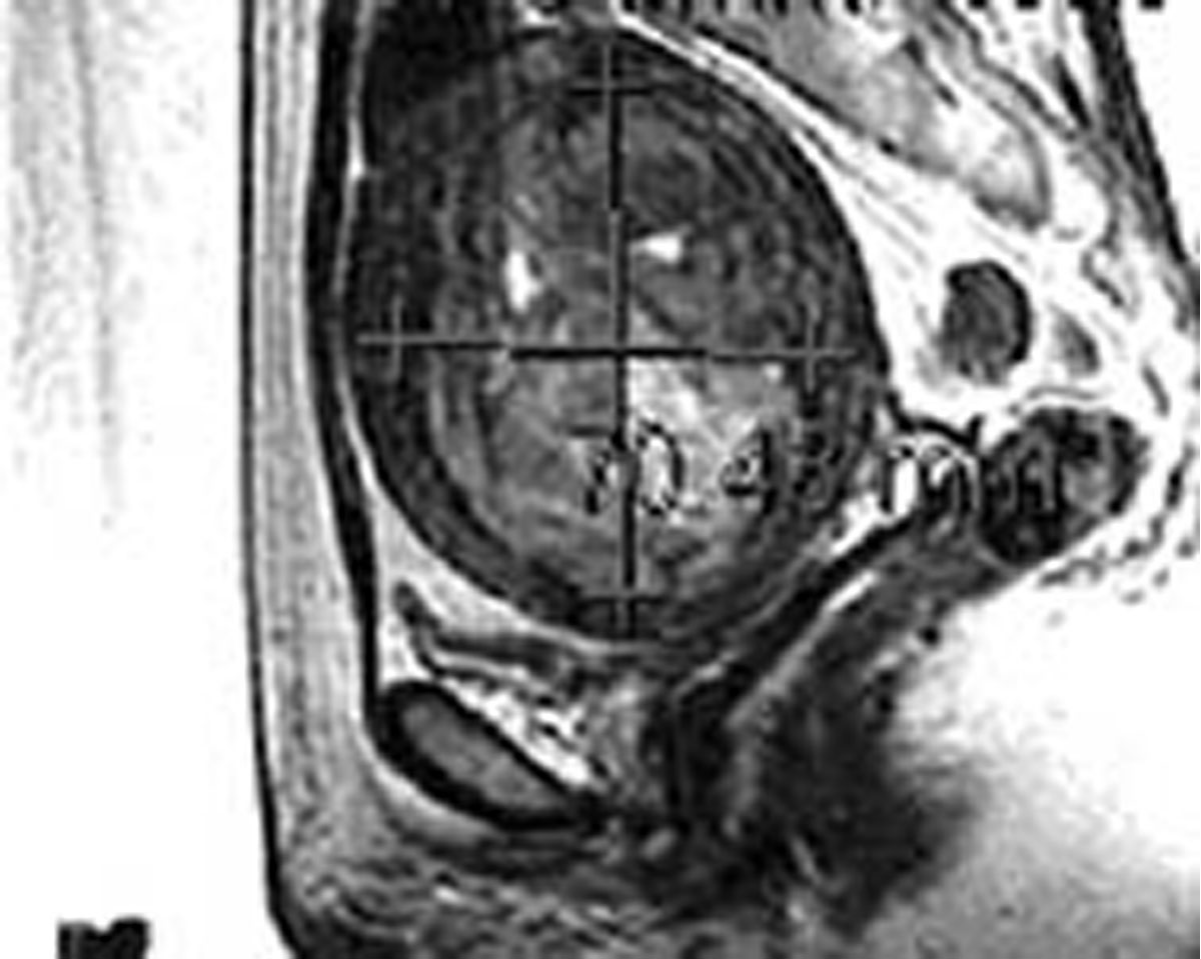
Sagittal T2-weighted image following 3 months of GnRH agonist therapy, showing a 281 cc fibroid representing a 40% reduction in volume.

**Figure 3:**
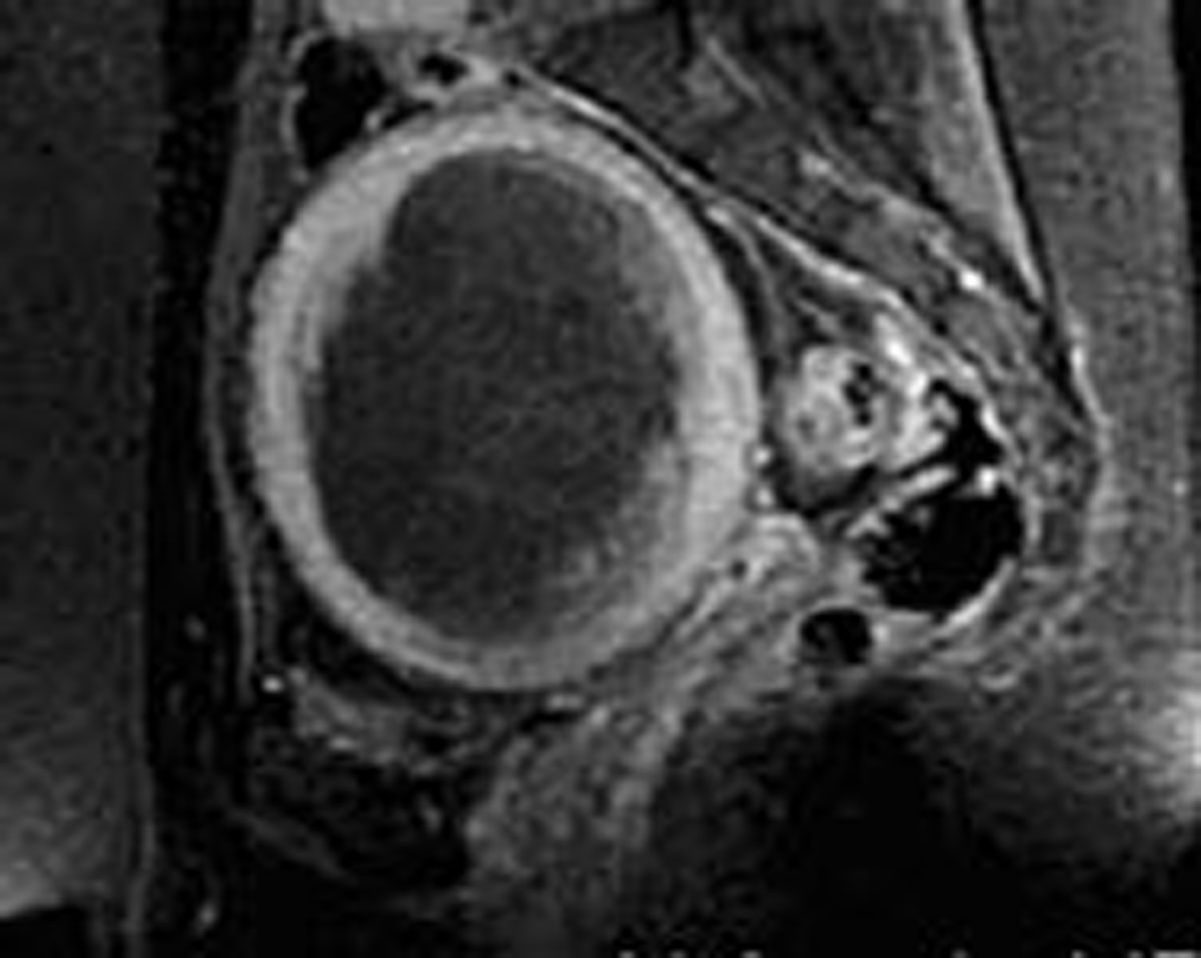
Sagittal post-contrast T1-weighted image following MRgFUS treatment demonstrates a non-perfused volume of 85%.

